# Spinal Biologics in Minimally Invasive Lumbar Surgery

**DOI:** 10.1155/2018/5230350

**Published:** 2018-04-05

**Authors:** Kevin Y. Chang, Wellington K. Hsu

**Affiliations:** Department of Orthopedic Surgery, Northwestern University Feinberg School of Medicine, 676 N. Saint Clair St., Suite #1350, Chicago, IL 60611, USA

## Abstract

As the use of minimally invasive spine (MIS) fusion approaches continues to grow, increased scrutiny is being placed on its outcomes and efficacies against traditional open fusion surgeries. While there are many factors that contribute to the success of achieving spinal arthrodesis, selecting the optimal fusion biologic remains a top priority. With an ever-expanding market of bone graft substitutes, it is important to evaluate each of their use as it pertains to MIS techniques. This review will summarize the important characteristics and properties of various spinal biologics used in minimally invasive lumbar surgeries and compare their fusion rates via a systematic review of published literature.

## 1. Introduction

Since Obenchain reported the first use of laparoscopic approach to lumbar spine discectomy in 1991, minimally invasive spine (MIS) surgery has evolved substantially over the years with the advances in robotics, imaging navigation systems, and endoscopy [1, 2]. Now more than ever, minimally invasive techniques are being applied in the treatment of a wide range of spine pathologies. With over 400,000 cases performed in the United States annually, spine fusion has become one of the most commonly performed and well-established treatment options for degenerative disorders, spine trauma, tumors, and structural deformities [3, 4].

When considering MIS fusion techniques, it is important to note that the biologic environment may differ not only from that of traditional open fusion surgeries but also among the different approaches. For example, in minimally invasive transforaminal interbody fusion (MIS-TLIF), there is limited access to the disc space for the complete removal of the disc due to surrounding neural structures [5]. On the other hand, for anterior and lateral approaches to interbody fusion such as oblique lateral interbody fusion (OLIF), extreme lateral interbody fusion (XLIF), and lateral lumbar interbody fusion (LLIF), the larger tubular access sits right on the disc space allowing for a more complete removal of the disc. This translates to an environment that is inherently more advantageous for fusion comparing to MIS-TLIF because of the greater surface area, enabling the placement of a bigger cage and more graft materials [5–9]. Nonetheless, selecting the ideal type of bone graft material for each approach is important to optimize efficacy and safety. This review will discuss and summarize the different types of fusion biologics used in minimally invasive lumbar fusions, their characteristics, properties, and efficacies.

## 2. Biology of Bone Regeneration, Repair, and Fusion

With a wide variety of fusion biologics available on the market, it is important to understand the basic characteristics of bone grafts in order to select the most appropriate graft material and maximize arthrodesis. The three fundamental properties of bone regeneration are osteogenesis, osteoinduction, and osteoconduction. Osteogenesis is defined as the formation of bone, and the “osteogenic” potential of a graft is its ability to provide appropriate cellular environment where the osteoprogenitor cells could survive transplantation and proliferate and differentiate into osteoblasts and osteocytes [10]. Osteoinductive properties of a graft refer to its ability to recruit immature cells and induce their proliferation and differentiation into bone-forming cells [11]. Lastly, osteoconduction describes the physical property of a graft material that allows bone cells such as osteoblasts and osteoclasts to attach, migrate, and grow in order to form viable bone [11, 12]. It is the interplay between all three of these properties of orthobiologics that leads to bone formation and regeneration.

Similarly, incorporation of bone grafts in spine fusion is highly dependent on these same principles. This process relies on many factors both systemically and locally between the graft and the host bed. Based on our current understanding, this process occurs in three distinct stages: (1) the inflammatory stage, (2) the repair stage, and (3) the late remodeling stage [12]. During the inflammatory stage (initial hours to days), osteoinduction drives chemotaxis and differentiation of host osteoprogenitor cells [78]. Inflammatory cells and fibroblasts are recruited through prostaglandin mediation into the graft where granulation and vascular tissues are formed. Osteoblasts also begin to form. The repair stage (weeks to months) is dominated by fibroblasts leading to the formation of a stroma supporting vascular ingrowth, as well as the deposition of a collagen matrix where osteoids are secreted and mineralization occurs [12]. This vascular growth also provides osteoclasts that could resorb the surfaces of the graft. Finally, the remodeling stage (months to years) is characterized by the restoration of the bone to its original structure and strength through mechanical stress placed on the bone over time [12]. During this time, the osteoconductive properties of the bone graft leads to the resorption and replacement of the graft tissue with new bone in a process called “creeping substitution” [78].

## 3. Method for Review of the Literature

**Figure 1:**
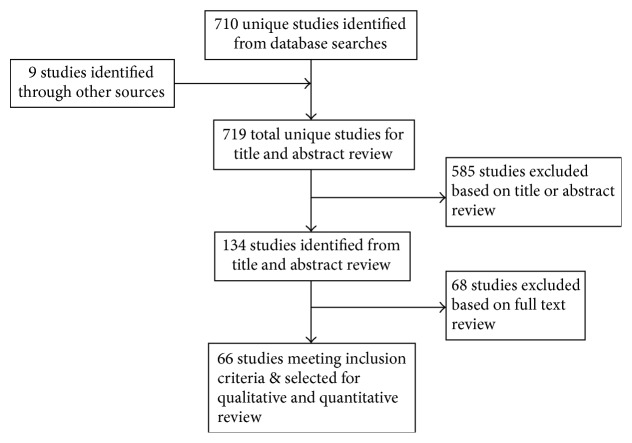
Systematic literature review and selection flowchart used to assess efficacies of various bone grafts.

MEDLINE was used to search for publications in English from 2005 through November 2017 with the keyword “minimally invasive” and combinations of the following: “posterior lumbar interbody fusion,” “transforaminal lumbar interbody fusion,” “anterior lumbar interbody fusion,” “extreme lateral interbody fusion,” “oblique lateral interbody fusion,” “direct lateral lumbar interobdy fusion,” and “lateral lumbar interbody fusion” (Figure 1). While this search yielded a total of 710 papers, additional articles were hand-retrieved from respective bibliographies. Inclusion criteria used were as follows: (1) clinical studies and case series with a minimum of 10 patients (2) adult patients with a minimum age of 18 years old, (3) studies explicitly stating the type of graft material used for fusion, and (4) studies reporting fusion rates by computed tomography (CT) or plain radiograph evaluation. For studies utilizing more than one type of bone graft, patients may be categorized into more than one group. A total of 66 studies met the inclusion criteria, and patients from each study were grouped based on the bone graft used (Table 3).

## 4. Bone Graft Materials

### 4.1. Autologous Graft: Iliac Crest Bone Graft and Local Bone Graft

An autologous bone graft is defined as bone harvested from one part of the body and transplanted to a different site in the same individual. Two of the most common sites where autologous bone grafts are derived from are the iliac crest or local spinous processes, lamina or facets. Iliac crest bone graft (ICBG) has historically been considered as the gold standard for spinal fusion due to its osteogenic, osteoinductive, and osteoconductive properties as well as its biocompatibility and mechanically stability [79, 80]. Furthermore, it also possesses minimal antigenic factors and no risk of disease transmission, eliminating risk of rejection [12]. However, this procedure has become less popular among spine surgeons in recent years due to the need for an additional surgical site, increased surgical time and blood loss, limited quantity, postoperative pain, and donor site complications [12, 79, 81]. These rates have been reported between 9 and 49% in the literature and ranged from minor superficial infections and seromas to major neurovascular injuries, deep infections, or herniation of abdominal contents through the harvest site [81, 82]. Of the six studies that met our inclusion criteria and utilized ICBG in MIS fusion procedures, two of them reported donor site complications. Ohtori et al. reported donor site pain at the location of the iliac crest in eight out of twelve patients [41]. In the prospective study by Peng et al., there were two cases (out of 29) of ICBG site infections that required wound debridement and antibiotic treatment postoperatively. Due to these drawbacks of ICBG, local bone harvest has become a more popular and viable option and, when available, is a common bone graft of choice in minimally invasive spine fusion procedures.

Regardless of where the autologous bone is harvested from, it is important to understand that the biomechanical properties of autografts vary between different types of autograft: cancellous versus cortical grafts [78, 80]. Cancellous bone grafts exhibit a much higher porosity than cortical grafts, which translates to significantly lower initial mechanical strength [78]. However, a higher porosity allows for easy revascularization and greater cellular diversity and biological activity allowing osteoblasts to form new bone. As remodeling occurs and the new graft is successfully incorporated, the mechanical strength of cancellous bone gradually increases [78, 80]. On the other hand, cortical bone is extremely dense and has a limited biologic profile. Even though it provides good initial strength and mechanical stability, vascular ingrowth is challenging. Therefore, osteoclasts must initiate the process of resorption first, leading to a transient decrease in mechanical strength, before revascularization could occur and new bone could be formed [80].

The efficacies of autografts have been widely reported in the literature. Based on our review, a total of 23 studies involving 1209 patients met our inclusion criteria and utilized autologous local bone without additional extenders and achieved a fusion rate of 91% (68–100%). Similarly, when ICBG was used alone, a fusion rate of 96% (90–100%) was calculated across 3 studies and 101 patients. Not surprisingly secondary to availability, no studies were found using only local autografts or combination of local and ICBG in lateral approaches. Ohtori et al. was the only study that utilized ICBG alone in OLIF with 12 patients, reporting a fusion rate of 90% [41].

### 4.2. Autologous: Bone Marrow Aspirate

Bone Marrow Aspirate (BMA) is a cell-based bone graft that can be harvested from either the iliac crest or the pedicle and utilized as an alternative to ICBG [83]. It provides an autogenic source of osteogenic precursor cells that have the potential to differentiate into osteoblasts to synthesize new bone tissues [79, 84]. While BMA also possesses other pluripotent mesenchymal stem cells and less donor site morbidity than ICBG, disadvantages include variability in processing, difficulty obtaining enough bone marrow with sufficient progenitors cells, and age-related decline in osteoprogenitor cells [79, 84–86].

Due to the lack of structural support and osteoconductive properties, BMAs are often combined with scaffolds including autografts, allografts, ceramics, and implants to achieve spinal fusion. BMAs can be harvested from the iliac crest or the vertebral body pedicles using needle suction prior to mixing with other bone graft materials [49–51, 53, 87]. For example, Nandyala et al. described filling an interbody cage with 5 mL of BMA from cannulated pedicles, local bone autograft, and rhBMP-2 in an MIS-TLIF [49]. In another application, Abbasi et al. combined tricalcium phosphate soaked in autologous BMAs aspirated from the pedicles using a Jamshidi needle before packing into the disc space in treatment of scoliosis with oblique lateral lumbar interbody fusion (OLLIF) [51]. Additionally, other studies have reported mixing BMAs harvested from the iliac crest with (1) *β*-tricalcium phosphate and hydroxyapatite (HA) in an extreme lateral interbody fusion (XLIF), (2) demineralized bone matrix (DBM) and rhBMP-2 packed in a polyether ether ketone (PEEK) cage in an oblique lateral interbody fusion (OLIF), and (3) local autograft, DBM, and cancellous allograft in XLIF procedure (Table 3) [50, 53, 87].

A total of 109 patients from 2 studies utilized BMAs as bone graft extenders (Table 1) in MIS-TLIF and achieved a fusion rate of 93% (93-94%) [49]. Furthermore, 271 patients were enrolled across 4 studies in retroperitoneal approaches to interbody fusions such as OLIF, XLIF, or OLLIF and achieved a fusion rate of 97% (93–100%) (Table 2).

### 4.3. Allograft

Human allograft is a type of bone graft that is transferred from an individual to another and may be obtained from either a cadaverous tissue or a donor patient following an operation such as the femoral head after a hip-replacement surgery. The mechanism of action of allografts is thought to be due to its osteoconductive properties as it provides a scaffold for bone formation and weakly osteoinductive depending on the preservation of growth factors after its processing [88]. It is important to note that these grafts do not possess any osteogenic potential because they do not contain any viable cells [89]. Compared to autologous bone graft, allografts are readily available at low costs and avoid additional donor site morbidity and mortality, making them a popular alternative [79]. Complications related to allograft use have been reported in the literature primarily concerning the potential risk of viral infection and disease transmission. Even though such transmission is extremely rare, there have been reported cases of human immunodeficiency virus (HIV), hepatitis B virus (HBV), hepatitis C virus (HCV), and certain bacterial infections [88, 89]. After proper screening and processing of the blood products, the reported risk of transmission is 1 in 63,000 for HBV, 1 in 100,000 for HCB, and less than 1 in 1,000,000 [90]. Not surprisingly, of all the studies included in this review that utilized allografts, there were no reports of allograft-related complications.

Allografts have rarely been used alone and almost always in combination with autografts, BMAs, DBMs, or rhBMP-2 for minimally invasive procedures (Table 3). From the 5 studies that used allograft as an extender to autografts in MIS-TLIF, a fusion rate of 96% was calculated (Table 1). Ahmadian et al. was the only study that used allograft alone in a lateral interbody fusion reporting a fusion rate of 90% (Table 2) [47].

### 4.4. Demineralized Bone Matrix

Demineralized Bone Matrix (DBM) is a human allograft-derived bone graft first introduced in 1991 as a substitute or additive to autologous bone grafts [91]. After its procurement from human cadaveric bone, it is treated with acid that removes the mineralized portion while maintaining the collagens (mostly type I with some types IV and X), the noncollagenous proteins, and certain growth factors (GF) [92]. The organic matrix (collagens and noncollagenous proteins) provides osteoconductive properties while the growth factors, which consist of bone morphogenetic proteins (BMP), transforming growth factor-*β*, and fibroblast growth factors among other GFs, contribute to its osteoinductive potential [93]. While the advantages of DBM include its unlimited availability and avoidance of an additional donor site comparing to autografts, its disadvantages have been well documented in the literature, most important of which are the significant variability in composition and efficacy not only between different products from different manufacturers but also within the same product from the same manufacturer [79, 94].

With over 35 commercially available products, DBM comes in a variety of different forms, such as dry powder, granules, injectable gels, putties, pastes, strips, flexible sheets, or blocks [92, 95]. Because DBM becomes particulate powder or fibers after initial processing that is difficult to manage clinically for surgeons, DBM is often combined with various biocompatible carriers such as sodium hyaluronate, carboxymethylcellulose, or glycerol into the form of moldable putty, thereby improving its handling properties [92].

Due to the lack of mechanical strength and ability to hold its structure intact, DBM is rarely used alone. The outcome of DBM use as an extender in lumbar fusion is widely reported in the literature. Kang et al. compared the efficacies of commercial DBM graft (Grafton™) with local autograft versus ICBG in patients who received a single-level posterior lumbar fusion and found that the fusion rates were comparable between the two groups (86% versus 92%) [96]. They also reported that the DBM group had a better clinical outcome with significantly less mean intraoperative blood loss and a higher physical function scores (though not statistically significant) at 24 months postoperatively [96]. In another study by Cammisa et al., 120 patients received ICBG implant on one side of the spine and DBM (Grafton)/autograft composite on the other side of the same patient. At 24-month follow-up, radiographic evidence showed similar fusion rates (ICBG, 54%; DBM, 52%), which suggests the efficacy of DBM as a bone graft extender is equally successful as autologous. Similar to the fusion rates reported by Kang et al., a fusion rate of 85% (77–97%) was calculated out of 264 patients (Table 1) in the 5 studies that we identified using DBM as an graft extender in MIS posterior interbody fusion procedures. In minimally invasive anterior and lateral interbody fusions, a total of 203 patients received DBM in addition to other biologics and achieved a fusion rate of 98% (97-98%), which is significantly higher than any bone graft material (*P* < 0.05) except for BMA as extender and rhBMP-2 alone for anterior/lateral approach to interbody fusion (Table 2).

### 4.5. Ceramics

Ceramics are calcium-based synthetic bone grafts that are used primarily as bone graft extenders because they possess only osteoconductive properties without any osteogenic or osteoinductive properties [89]. They are made from a process called “sintering,” which essentially uses high temperatures to force various chemical compounds to bond together [97]. Because they are manufactured through a controlled chemical process, their forms, compositions, porosities, biodegradability, and other characteristics can be modified. Some advantages of ceramics include immediate strength, no risk of disease transmission, unlimited supply, long-shelf life, and availability in many different forms and compositions, while its disadvantages include its brittleness and lack of cortical stability [79, 89].

Materials that are commonly found in ceramics include hydroxyapatite (HA), tricalcium phosphate (TCP), calcium phosphate, and calcium sulfate. Commercially, they come in various preparations and are either found alone or in compositions with one another to provide different properties of biodegradability, porosity, and utility [98]. One of the most important properties to understand when using ceramics in orthopedic procedures is the porosity of different types of ceramics and how it contributes to the biomechanical strength of the graft. *β*-TCP has a higher porosity and pore size than HA, leading to a larger surface area and lower compressive strength. However, this allows for a higher biologic response, faster osteoclastic resorption, and the subsequent osteoblastic bone formation. HA, on the other hand, is more dense and undergoes a slow resorption but provides a much stronger biomechanical stability [10]. Considering these factors, HA and *β*-TCP are often modified and combined with each other to enhance the functionality and take advantage of strengths of each material [10, 99]. Multiple studies have also reported using HA, *β*-TCP, or calcium phosphate alone as a supplement to an osteoinductive source in anterior/lateral or posterior approach to MIS fusion [13, 51, 59].

A total of 4 studies that met our inclusion criteria used ceramics either alone or as bone graft extenders. In the 3 studies that used ceramics as extenders to osteoinductive local autograft, a fusion rate of 86% (83–88%) was observed (Table 1), which is consistent with the literature for open spine procedures [98]. In the only study that reported using ceramics alone, Alimi et al. reported a fusion rate of 76.3% in MIS-TLIF when silicate substituted calcium phosphate (Si-CaP) was used [64]. Despite the lower fusion rate in MIS-TLIF, the authors concluded that Si-CaP alone is still a viable option as a bone graft substitute because the overall fusion rate was 82.9% when all fusion procedures in the study were considered [64]. In retroperitoneal approaches, the collective fusion rates are 95% (80–100%) and 88% (80–100%) corresponding to ceramics used as an extender and alone, respectively (Table 2). The lower fusion rate seen with ceramics alone may be due to the lack of osteoinductive agents.

### 4.6. Bone Morphogenetic Protein 2

Bone morphogenetic proteins (BMPs) are endogenous, soluble proteins that are members of the transforming growth factor-*β* (TFG-*β*) family found in the human body [79]. They are growth factors that are involved in a myriad of cellular responses including the differentiation, maturation, and proliferation of mesenchymal stem cells (MSCs) into mature cartilage- and bone-forming cells [79, 100]. BMPs are thought to possess osteoinductive properties by binding serine-threonine kinase receptors on cell surfaces and activating Smad (Smad1/5) complex proteins, leading to their translocation into the nucleus and inducing expression of genes related to osteoblast phenotypes [101]. After its discovery by Marshall Urist through partially purified extract of DBM in 1965, over 20 BMPs have since been identified to play an active role in fracture healing and formation of new bone [102, 103]. With the advent of genomic technology, they have become commercially available in the form of recombinant proteins and have become one of the most popular bone graft substitutes in MIS fusion procedures [103].

However, its limitations include a potential side effect profile of seroma/hematoma formation, prevertebral swelling, radiculitis, retrograde ejaculation, osteolysis, and heterotopic ossification [79, 100, 104]. In a retrospective review of 610 patients who has underwent MIS-TLIF, Singh et al. have shown that heterotopic ossification and osteolysis are both complications associated with rhBMP-2 use [104]. Heterotopic ossification is characterized by bone formation at an abnormal anatomical site, usually the soft tissue, where bone normally does not exist. Osteolysis, on the other hand, describes the opposite process where there is a pathological destruction of bone tissue. In addition, rhBMP-2 use in the cervical spine has been linked to life-threatening complications such as airway edema and dysphagia, leading to the FDA issuing a warning its use in cervical spine procedures in 2008 [105–107]. Furthermore, there have been multiple studies, most notably the study published from the Yale University Open Data Access (YODA) collaboration with Medtronic, linking the use of rhBMP-2 to an increase in incidence of cancer [108, 109]. However, two large-scale retrospective studies with a combined cohort of over 600,000 patients concluded that there is no evidence of increased malignancy with the use of rhBMP-2 [110, 111]. From the 8 studies that met our inclusion criteria utilizing rhBMP-2 in MIS-TLIF, Nandyala et al. documented a case of rhBMP-2 related neuroforaminal bone growth and cage migration/osteolysis and Siddiqui et al. reported two cases of bone overgrowth with radiculopathy, a case of osteolysis, and two cases of radiculitis [49, 70].

Surgical technique involving rhBMP-2 (INFUSE) in MIS procedures have been described within the implant, disc space, or both. For example, Tsahtsarlis and Wood and Rouben et al. both described inserting a cage filled with local autograft and rhBMP-2 after end-plate preparation, whereas Wang and Grossman and Siddiqui et al. packed rhBMP-2 into the anterior disc space [69–72]. Two other MIS-TLIF studies inserted local autograft and rhBMP-2 soaked collagen sponge anteriorly within the disc space as well as inside the interbody implant (Table 3) [73, 74]. While the site of rhBMP-2 insertion can vary based on surgeon preference and the pathologies, the relative dose of rhBMP-2 also differed. Of all the MIS-TLIF studies we included, Tsahtsarlis and Wood, Siddiqui et al., and Nandyala et al. reported using 1.4 mg, 4.2 mg, and either 4.2 mg or 12 mg, respectively [49, 70, 71]. Notably, the majority of these studies used CT scans as the method of evaluation for fusion during their postoperative follow-ups (Table 3).

In patients utilizing rhBMP-2 as a bone graft substitute in addition to autografts or allografts in minimally invasive posterior interbody fusion approaches, a collective fusion rate of 95% (65–100%) was reported (Table 1). This rate is consistent with those reported by the meta-analysis performed by Parajón in MIS-TLIF [112]. In the only study that used rhBMP-2 alone, Dahdaleh et al. concluded that there was no significant difference in unilateral and bilateral instrumentation in patients undergoing MIS-TLIF with fusion rates of 93.8% and 95%, respectively [77]. The combined fusion rate for those two groups is 94.4% (Table 1). While these fusion rates are robust, it is not statistically significant compared to ICBG alone, autograft plus allograft, and BMA in posterior approaches. When rhBMP-2 is used in anterior/lateral interbody fusion techniques alone and with other fusion biologics, fusion rates of 97% (96–100%) and 97% (80–100%) were calculated, respectively (Table 2).

## 5. Conclusion

With an ever-expanding arena of fusion biologics and bone graft materials, it is important to understand each of their properties, advantages, and disadvantages, as well as their applications. While ICBG remains the gold standard for spine fusion surgeries, local autologous bone graft is by far the predominant bone graft material utilized in minimally invasive posterior approach to interbody fusion. In anterior/lateral techniques, ceramics and rhBMP-2 are the most common, likely due to the need for the mechanical properties of ceramics as well as rhBMP-2s strong osteoinductivity in treating more severe structural deformities. Overall, fusion rates were high with over 90% and 95% calculated for MIS posterior and MIS anterior/lateral approaches to interbody fusion, respectively, regardless of bone grafts used.

## Figures and Tables

**Table 1:** Fusion rates for various bone grafts in posterior approaches to minimally invasive interbody fusions.

Bone grafts material	Posterior approaches to MIS interbody fusion			
	Number of studies	Number of patients	Fusion rate (%)	Range (%)
Autograft (local and iliac crest)	2	29	86	83–91
Autograft (local) alone	23	1209	91	68–100
Autograft (iliac crest) alone	3	101	96^a^	90–100
Autograft + allograft	5	178	96^a^	92–100
BMA (as extender)	2	109	93^a^	93-94
DBM (as extender)	5	264	85	77–97
Ceramics (as extender)	3	217	86	83–88
Ceramics alone	1	57	76	76
rhBMP-2 (with another bone graft)	7	486	95^a^	65–100
rhBMP-2 alone	1	36	94^a^	94

^a^There are no significant differences in fusion rates among these groups; however, they do demonstrate significantly higher fusion rates when compared to the rest (*P* < 0.05).

**Table 2:** Fusion rates for various bone grafts in anterior/lateral approaches to minimally invasive interbody fusions.

Bone graft materials	Anterior/lateral approaches to mis interbody fusion			
	Number of studies	Number of patients	Fusion rate (%)	Range (%)
Autograft (iliac crest) alone	1	12	90	90
Allograft alone	1	40	90	90
BMA (as extender)	4	271	97^b^	93–100
DBM (as extender)	2	203	98^a^	97-98
Ceramics (as extender)	5	149	95	80–100
Ceramics alone	5	206	88	80–100
rhBMP-2 (with another bone graft)	7	308	96^b^	80–100
rhBMP-2 alone	2	122	97^b^	96–100

^a^DBM (as extender) demonstrates significantly higher fusion rates than all bone grafts (*P* < 0.05), except for BMA (as extender) and rhBMP-2 alone. ^b^These three types of bone grafts (BMA as extender, rhBMP-2 with another bone graft, and rhBMP-2 alone) are no different from each other and DBM in terms of fusion rate but significantly better than the rest (*P* < 0.05).

**Table 3:** Published peer-reviewed studies utilizing various bone grafts in MIS interbody fusion procedures identified through our literature search.

Bone graft material	Author and year	Type of procedure	Type of material used in disc space	Type of material used in the cage	Method of fusion evaluation	Number of patients	Fusion rate (%)
Autograft (local and ICBG)	Yoo et al. 2015 [13]	MIS-TLIF	NA	Autograft (local + ICBG)	X-ray + CT	11	90.9
	Schizas et al. 2009 [14]	MIS-TLIF	Autograft (local + ICBG)	Autograft (local + ICBG)	X-ray	18	83.3

Autograft (local) alone	Zhang et al. 2017 [15]	MIS-TLIF	NA	Autograft (local)	X-ray + CT	26	100
	Rajakumar et al. 2017 [16]	MIS-TLIF	NA	Autograft (local)	X-ray	36	97.2
	Yang et al. 2017 [17]	MIS-TLIF	Autograft (local)	Autograft (local)	NA	21	85.7
	Huang et al. 2017 [18]	MIS-TLIF	Autograft (local)	Autograft (local)	X-ray	58	87.9
	Choi et al. 2017 [19]	MIS-TLIF	Autograft (local)	Autograft (local)	CT	44	95.2
	Choi et al. 201 7[19]	MIS-TLIF	Autograft (local)	Autograft (local)	CT	40	96.6
	Lv et al. 2017 [20]	MIS-TLIF	NA	Autograft (local)	CT	50	96.0
	Fan et al. 2016 [21]	MIS-TLIF	NA	Autograft (local)	X-ray	41	92.7
	Fan et al. 2016 [21]	MIS-TLIF	NA	Autograft (local)	X-ray	37	81.1
	Tian and Liu 2016 [22]	MIS-TLIF	Autograft (local)	NA	CT	47	90.0
	Tian and Liu 2016 [22]	W-TLIF	Autograft (local)	NA	CT	50	88.9
	Fan et al. 2016 [23]	MIS-TLIF	Autograft (local)	Autograft (local)	X-ray	24	91.7
	Fan et al. 2016 [23]	MIS-TLIF	Autograft (local)	Autograft (local)	X-ray	21	85.7
	Yang et al. 2015 [24]	MIS-TLIF	Autograft (local)	Autograft (local)	X-ray	50	88.0
	Liu et al. 2015 [25]	MIS-TLIF	Autograft (local)	NA	X-ray	42	81.0
	Liu et al. 2015 [25]	MIS-TLIF	Autograft (local)	NA	X-ray	42	95.2
	Lee et al. 2016 [26]	MIS-TLIF	NA	Autograft (local)	X-ray + CT	27	81.5
	Chen et al. 2015 [27]	MIS-TLIF	Autograft (local)	NA	CT	42	100
	Chen et al. 2015 [27]	MIS-TLIF	Autograft (local)	NA	CT	36	100
	Kim et al. 2015 [28]	MIS-TLIF	NA	Autograft (local)	CT	18	88.9
	Kim et al. 2015[28]	MIS-TLIF	NA	Autograft (local)	CT	23	87.0
	Zhang et al. 2015 [29]	MMI-TLIF	Autograft (local)	NA	NA	49	81.7
	Gu et al. 2015 [30]	MIS-TLIF	Autograft (local)	Autograft (local)	X-ray	35	94.3
	Gu et al. 2015 [30]	MIS-TLIF	Autograft (local)	Autograft (local)	X-ray	39	94.9
	Shen et al. 2014 [31]	MIS-TLIF	Autograft (local)	NA	CT	31	96.8
	Shen et al. 2014 [31]	MIS-TLIF	Autograft (local)	NA	CT	34	100.0
	Gu et al. 2014 [32]	MIS-TLIF	Autograft (local)	Autograft (local)	X-ray	44	93.2
	Choi et al. 2013 [33]	MIS-TLIF	NA	Autograft (local)	X-ray + CT	26	84.6
	Choi et al. 2013 [33]	MIS-TLIF	NA	Autograft (local)	X-ray + CT	27	96.3
	Sonmez et al. 2013 [34]	MIS-TLIF	Autograft (local)	Autograft (local)	X-ray	10	80.0
	Sonmez et al. 2013 [34]	MIS-TLIF	Autograft (local)	Autograft (local)	X-ray	10	90.0
	Wang et al. 2014 [35]	MIS-TLIF	Autograft (local)	NA	X-ray + CT	43	97.7
	Kasliwal and Deutsch 2012 [36]	MIS-TLIF	Autograft (local)	Autograft (local)	CT	40	67.5
	Scheufler et al. 2007 [37]	MIS-TLIF	Autograft (local)	Autograft (local)	CT	46	93.5

Autograft (ICBG) alone	Serban et al. 2017 [38]	MIS-TLIF	Autograft (ICBG)	NA	CT	40	90
	Fan et al. 2010 [39]	MIS-TLIF	Autograft (ICBG)	Autograft (ICBG)	X-ray	32	100
	Peng et al. 2009 [40]	MIS-TLIF	Autograft (ICBG)	NA	X-ray	29	100
	Ohtori et al. 2015 [41]	OLIF	NA	Autograft (ICBG)	CT	12	90

Autograft + allograft	He et al. 2017 [42]	MIS-TLIF	Autograft (local) + Allograft	Autograft (local) + Allograft	CT	42	92.9
	Lee et al. 2016 [43]	MIS-TLIF	NA	Autograft (local) + Allograft	X-ray	38	100
	Lim and Kim 2013 [44]	MIS-TLIF	Autograft (local) + Allograft	NA	X-ray + CT	19	100
	Kim et al. 2011 [45]	MIS-TLIF	Autograft (local) + Allograft	Autograft (local) + Allograft	X-ray	56	96.4
	Jang and Lee 2005 [46]	MIS-TLIF	NA	Autograft (local +/− ICBG) + Allograft	X-ray	23	91.7

Allograft alone	Ahmadian et al. 2015 [47]	MIS-LIF	Allograft (unclear interbody or in cage)		X-rays +/− CT	40	89.7

BMA (as extender)	Hawasli et al. 2017 [48]	MIS-TLIF	Allograft + bma	Autograft (local) + BMA	X-ray +/− CT	44	93.2
	Nandyala et al. 2014 [49]	MIS-TLIF	Autograft (local) + rhBMP-2 + BMA	Autograft (local)	CT	65	93.8
	Woods et al. 2017 [50]	OLIF	DBM + BMA + rhBMP-2	DBM + BMA + rhBMP-2	CT	137	97.9
	Abbasi et al. 2017 [51]	OLLIF	*β*-TCP + BMA	NA	NA	24	100
	Rodgers et al. 2012 [52]	XLIF	Formagraft (*β*-TCP + HA) + BMA (unclear interbody or in cage)		X-ray + CT	44	93.2
	Rodgers et al. 2010 [53]	Mini-ALIF XLIF	Autograft (local) + Allograft + DBM + BMA		CT	66	97.0

DBM (as extender)	Choi et al. 2016 [54]	MIS-TLIF	Autograft (local) + DBM	Autograft (local) + DBM	CT	21	95.2
	Kuo et al. 2016 [55]	MIS-TLIF	NA	Autograft (local) + DBM	X-ray + CT	22	77.3
	Park et al. 2014 [56]	MIS-TLIF	Autograft (local) + DBM	NA		83	80.7
	Lee et al. 2012 [57]	MIS-TLIF	Autograft (local) + DBM	NA	X-ray	72	97.0
	Park et al. 2011 [58]	MIS-TLIF	Autograft (local) + DBM	NA	CT	66	77.3
	Woods et al. 2017 [50]	OLIF	DBM + BMA + rhBMP-2	DBM + BMA + rhBMP-2	CT	137	97.9
	Rodgers et al. 2010 [53]	Mini-ALIF/XLIF	Autograft (local) + Allograft + DBM + BMA		CT	66	97.0

Ceramic (as extender)	Yoo et al. 2014 [59]	MIS-TLIF	NA	Autograft (local) + HA	X-ray + CT	92	87.0
	Yoo et al. 2015 [13]	MIS-TLIF	Autograft (local) + HA	Autograft (local)	X-ray + CT	49	87.8
	Yoo et al. 2015 [13]	MIS-TLIF	Autograft (local) + HA	Autograft (local)	X-ray + CT	28	85.7
	Lin and Chiang 2014 [60]	MIS-TLIF	Autograft (local) + SinboneHT (60% HA, 40% TCP)	NA	X-ray	48	83.3
	Malham et al. 2017 [61]	XLIF	NA	Mastergraft (*β*-TCP + HA) + rhBMP-2	CT	21	95.2
	Malham et al. 2017 [61]	XLIF	NA	Mastergraft (*β*-TCP + HA) + rhBMP-2	CT	19	80.0
	Abbasi et al. 2017 [51]	OLLIF	*β*-TCP + BMA	NA	NA	24	100
	Waddell et al. 2014 [62]	LLIF	NA	Mastergraft (*β*-TCP + HA) + rhBMP-2	CT	21	98.0
	Rodgers et al. 2012 [52]	XLIF	Formagraft (*β*-TCP + HA) + BMA (unclear interbody or in cage)		X-ray + CT	44	93.2
	Dakwar et al. 2010 [63]	XLIF	NA	TCP + HA + rhBMP-2	X-ray +/− CT	20	100.0

Ceramics alone	Alimi et al. 2017 [64]	MIS-TLIF	NA	Si-CaP	CT	57	76.3
	Parker and Malham 2017 [65]	XLIF	NA	AttraX (*β*-TCP + HA)	CT	25	80.0
	Alimi et al. 2017 [64]	XLIF	NA	Si-CaP	CT	44	82.1
	Berjano et al. 2015 [66]	XLIF	NA	AttraX (*β*-TCP + HA)	X-ray + CT	13	83.0
	Berjano et al. 2015 [66]	XLIF	NA	Calcium Triphosphate	X-ray + CT	35	89.0
	Marchi et al. 2013 [67]	XLIF	Calcium Phosphate (unclear interbody or in cage)		CT	74	91.0
	Pimenta et al. 2013 [68]	XLIF	Si-CaP (unclear interbody or in cage)		X-ray + CT	15	100.0

rhBMP-2 (with another graft)	Wang and Grossman 2016 [69]	MIS-TLIF	rhBMP-2	Allograft	X-ray	10	87.5
	Siddiqui et al. 2016 [70]	MIS-TLIF	Autograft (local) + rhBMP-2	Autograft (local)	X-ray	148	98.5
	Nandyala et al. 2013 [49]	MIS-TLIF	Autograft (local) + rhBMP-2 + BMA	Autograft (local)	CT	65	93.8
	Tsahtsarlis and Wood 2012 [71]	MIS-TLIF	NA	Autograft (local) + rhBMP-2	CT	34	97.1
	Rouben et al. 2011 [72]	MIS-TLIF	NA	Autograft (local) + rhBMP-2	X-ray + CT	169	96.0
	Park and Foley 2008 [73]	MIS-TLIF	Autograft (local) + rhBMP-2	Autograft (local) + rhBMP-2	CT	40	100
	Deutsch and Musacchio Jr. 2006 [74]	MIS-TLIF	Autograft (local) + rhBMP-2	Autograft (local) + rhBMP-2	CT	20	65.0
	Malham et al. 2017 [61]	XLIF	NA	Mastergraft (*β*-TCP + HA) + rhBMP-2	CT	21	95.2
	Malham et al. 2017 [61]	XLIF	NA	Mastergraft (*β*-TCP + HA) + rhBMP-2	CT	19	80.0
	Woods et al. 2017 [50]	OLIF	DBM + BMA + rhBMP-2	DBM + BMA + rhBMP-2	CT	137	97.9
	Ahmadian et al. 2015 [47]	MIS-LIF	Allograft + rhBMP-2 (unclear interbody or in cage)		X-rays +/− CT	19	100.0
	Waddell et al. 2014 [62]	LLIF	NA	Mastergraft (*β*-TCP + HA) + rhBMP-2	CT	21	98.0
	Castellvi et al. 2014 [75]	LLIF	NA	Allograft + rhBMP-2	CT	36	100.0
	Dakwar et al. 2010 [63]	XLIF	NA	TCP + HA + rhBMP-2	X-ray +/− CT	20	100.0
	Aryan et al. 2008 [76]	AxiaLIF	Autograft (local) + rhBMP-2		X-ray +/− CT	35	91.4

rhBMP-2 alone	Dahdaleh et al. 2013 [77]	MIS-TLIF	NA	rhBMP-2	X-ray	20	95.0
	Dahdaleh et al. 2013 [77]	MIS-TLIF	NA	rhBMP-2	X-ray	16	93.8
	Parker and Malham 2017 [65]	XLIF	NA	rhBMP-2	CT	107	96.3
	Pimenta et al. 2013 [68]	XLIF	rhBMP-2 (unclear interbody or in cage)		X-ray + CT	15	100.0

MIS, minimally invasive spine; TLIF, transforaminal lumbar interbody fusion; ALIF, anterior lumbar interbody fusion; XLIF, extreme lateral interbody fusion; OLIF, oblique lumbar interbody fusion; OLLIF, oblique lateral lumbar interbody fusion; ICBG, iliac crest bone graft; BMA, bone marrow aspirate; DBM, demineralized bone graft; rhBMP-2, recombinant morphogenetic protein 2; HA, hydroxyapatite; TCP, tricalcium phosphate; Si-CaP, silicated calcium phosphate; CT, computed tomography; NA, not available.
